# Prof. Noel's Reply to Prof. Judd


**Published:** 1870-06

**Authors:** 


### ARTICLE III.

### *Prof. Noel's Reply to Prof. Judd.**

The April No. of the *Missouri Dental Journal* has a short criticism upon an article published in the Feb. No. of the *American Journal of Dental Science;* the article in question was an abstract of a lecture delivered before the class at the Baltimore College of Dental Surgery, and embraced the most recent views as regards the histology of enamel and dentine.

As the author of this piece, I wish to explain my views more clearly, and endeavor to remove the misapprehension under which I think my *critic* is laboring. I regret that I should have been deficient in lucidness, and while adhering most tenaciously to the opinions already advanced, I am ready at any moment to yield them, *provided*, they can be proven false and better ones substituted for them.

In this lecture I asserted, that, "Fresh Dentine has no tubuli but has uncalcified fibrillæ solid, or nearly solid;" also "that the tubuli found in the dry tooth, or dead and dry tooth, are formed by the desiccation and evaporation of these dentinal fibrillæ, and have no existence in the living tooth."

The writer in the *Missouri Journal* makes the following comment upon those two statements: "*We must confess that we do not exactly understand why a set of hollow rods should be converted into fibrillæ, and declared not to be*

---

***See editorial for Prof. Judd's, review of Prof. Noel's Article, on "Enamel and Dentine."**

*tubuli, because they are filled with a fluid or a soft solid matter. Fill a hard tube with water, or with any other substance that does not blend with it and make a part of the solid substance itself, and it will not change the tube into a fibre, or in any way destroy its identity.*"

The first portion of the quotation is a "petitio principii" and in assuming the existence of the "hollow rods" the writer has taken an unproved position, and reasons from it as if he had demonstrated the actual existence of the "hollow rods" in the fresh dentine. And as to converting "hollow rods" into fibrillæ, I do not think any one ever attempted such a feat—my language was most thoroughly misconstrued if such an idea was derived from it.

*The Dentine Organ.* I asserted that the Dentine Organ, prior to calcification, consisted of fibrillæ more or less branching and arborescent; that it began near the inner surface of the enamel organ and terminated by blending in organic continuity with the pulp tissue; that these fibrillæ occupied the entire dentine organ and there was no interfibrillæ substance whatever; that in most of the fibrillæ the entire germinal or nuclear matter, changed into formed material, as soft semi-solid fibrillæ substance; but in some few, or comparatively a smaller number, the germinal or nuclear matter in the central axis of the fibrillæ remained *for a time* unchanged, or it might remain *permanently* as germinal matter; but there was a possibility of this central axis of germinal matter changing to formed material at any time, that is it might become fibrillæ substance as all the germinal matter of the solid fibrillæ, had done before it; once changed and the fibrillæ would be identical in every respect with the other solid fibrillæ; that this change might take place after the tooth was calcified, for the surrounding fibrillæ might calcify and leave the incompletely formed fibrillæ uncalcified; now these uncalcified fibrillæ would be surrounded by *dentine* resulting from the direct calcification of contiguous fibrillæ.

The fibrillæ yet uncalcified, and having a periphery of formed material and central axis of nuclear matter, may remain for years in this condition; or they may have the formed periphery calcified, leaving only a central axis of nuclear matter with or without a thin stratum of formed material between the germinal matter and calcifying periphery; again, the whole central axis of nuclear or germinal matter may change gradually to formed material and as formed material may undergo calcification and become dentine; thus we might have solid dentine on the exact line once occupied by the axis cylinder of nuclear or germinal matter. In such instances the germinal matter (*central*) changes to formed material, as the formed material (*peripheral*) calcifies and becomes dentine. This may take place at any time from youth to old age, and is the true explanation of the "condensation of dentine." That is to say—Dentine in adult life may become denser—harder, and better by calcification of these fibrillæ; *all dentine is calcified fibrillæ and all fibrillæ are liable to become calcified and thus form dentine; therefore at all times dentine is being formed and fibrillæ are being more or less completely transformed into it; there is no inter fibrillæ substance, which calcifying becomes dentine, but dentine results from a direct calcification of the fibrillæ themselves, from the line of conjunction of enamel and dentine to the periphery of the pulp.* The solid portions of the fibrillæ and the connective tissue of the pulp—the central axis of nuclear matter and the germinal or nuclear matter of the connective tissue blend so intimately, that the organic continuity seems unbroken upon the periphery of the pulp; and this pulp periphery is a purely arbitrary thing with no fixed limit, for just as the fibrillæ in the dentine may calcify slowly, so may the terminal ends (of all the fibrillæ already calcified to form dentine) slowly calcify and encroach upon the pulp, giving an increase of dentine by exactly the amount of pulp lost.

There are no canals, but semi-solid fibrillæ in fresh dentine, and these fibrillæ in the living tooth may calcify and give solid dentine; the calcified and the uncalcified portions of a fibrilla do not remain upon fixed lines, but the calcification has a tendency to slowly encroach upon the fibrilla and may even completely transform it into dentine. In a living tooth, therefore, there can be no dentinal tubes in the ordinary acceptation of the phrase, and the tubes of the dry tooth depend upon the desiccation and evaporation of the fibrillæ which here remain uncalcified, and the argument drawn from the "hard tube filled with water," &c., &c., does not apply to the case in question as all must at once see, for the fibrillæ need only the mineral deposition to become dentine ; and the surrounding dentine is nothing more than this mineral deposition in fibrillæ.

I also asserted that "Enamel and Dentine are not vitalized tissues so far as the calcified portions are concerned—enamel is dead, perfectly dead and so is the calcified portion of dentine (or dentine proper)." To this the writer objects, and thinks my use of the term "organic base," to represent the enamel and dentine organs prior to calcification gives proof of their being vitalized tissues after calcification. This will depend upon *his* definition of a "vitalized tissue," —I used the phrase in the sense that recent physiological writers have adopted, viz: of "*changing under vital forces*"—of exhibiting the three forces of organic life (1) assimulation, (2) propagation, (3) developement; the *organs* are organic products,—products of vital action and when calcified become enamel and dentine, but by the very act of calcification they lose their position with soft structures and take a perfectly distinct relation to the system, subserving the purest kind of physical purpose. The calcified enamel never changes and the calcified dentine never changes, *save in obedience to physical forces ;* bone does change, is constantly changing, is being renewed and transformed from birth until death, certainly until extreme old age ; it changes in obedience to the forces of organic life, enamel and dentine do not. Enamel filed or cut does not renew itself, dentine cannot renew itself, and can only condense in virtue of its fibrillæ ; metallic pins, rings &c., placed in bone are found after varying periods, to have changed their positions with respect to each other and with respect to the bone, because the bone has undergone vital changes; but do fillings of gold ever become imbedded in enamel or dentine, or are they ever covered by growths of new enamel or new dentine?

The hair and the nails,—the fragments we are accustomed to clip off and throw away, belong to the same class of structures as the teeth; they have an "organic base" and are equally as dead as enamel so far as the usual signs of vitality are concerned; the central axis of the hair, near the hair follicle may have masses of germinal matter and may exhibit a feeble degree of vital power, but at the extreme end it has only a physical form, and like the nail has no sensibility and undergoes no change save in obedience to physical forces.

If enamel be alive and if it can undergo any change whatever in virtue of its organic base, pray what is the change? Certainly not renewal, for filling the root with gold and taking off every possibility of vascular and nervous supply does not alter it at all; nor does it alter the calcified dentine. Are there any *soft structures* in the body which can be for an indefinite period of time, say years, deprived of all vascular and nervous supply and yet live? Now "*the general law which dominates over all other soft tissues*" to use the writers own language, proves conclusively that enamel and dentine are not soft tissues. Or does killing the pulp and removing it, also kill the enamel and dentine? if so, what are the evidences of their death? They remain for years apparently unchanged, and often exhibit decay with all the phenomena incident upon that process except pain; if enamel undergoes any change in obedience to vital force what is the change, since interstitial nutritive changes are certainly not to be found in it? and the pain in decay is dependent upon the dentinal fibrillæ and not upon the enamel; enamel in itself is perfectly insensible, it has neither nerves, nor blood vessels—nor fibrillæ; if enamel ever gives evidence of sensibility it must be in extremely rare cases, and only where the dentinal fibrillæ have been prolonged into the enamel, or rather into the enamel organ and have escaped calcification, thus giving an unbroken line from enamel to pulp.

My position is wrong also, says this critic, because the assertion that enamel and dentine having an "*organic base*," which calcified will remain "*unchanging and unchanged during a long term of years, militates against that higher law which ordains that constant change is stamped upon every atom of matter throughout the universe;*" now that phrase is rather a pretty one, but has not the least particle of force as an argument. I contend that enamel does not change in obedience to "*vital force*" and therefore is not a vitalized structure; that it may and does change in obedience to physical forces I admit, but that only confirms the assertion that it is "*dead;*" that as far as vital force is concerned, once calcified, and it is a mere mineral and obeys physical and chemical, but is not amenable to any of the laws of vital force.

Every atom of matter in the universe *may be under a law* of "incessant change," but if the changes are to us *inappreciable*, I contend that it is not proven. Before me is an old glass ink stand I have had for years, it is evidently composed of atoms of glass and they may be "constantly changing" but I cannot appreciate these changes; my upper central incisors have been filed apart, it was performed some ten years ago, the atoms of enamel like the atoms of glass, may be constantly changing but I cannot appreciate the slightest change in one any more than I can in the other, and how shall I know that they exist?

I have a friend travelling in Europe, he writes to me that he saw at a Cathedral, a molar tooth said to have belonged to one of the apostles or bishops of the "Early Church;"—evidently some hundreds of years old;—now perhaps the "organic base" of the enamel of that tooth has been undergoing "constant change" for the last 1800 years, or at any rate a great number of years, but my friend failed to appreciate it, for he said, "it was a fine tooth," "in a state of excellent preservation."

Finally—this writer says—*If we were asked to furnish evidence that enamel was not dead—perfectly dead—we would point to the fact, that it is many times exceedingly sensitive to the touch and that sensibility is one of the most certain signs of vitality wherever it exists. Can any one give as good a reason for supposing it dead?*

1. It is an assertion unproven, to say that enamel in *itself* is sensitive, it has neither nerves nor fibrillæ.

2. If *sensitiveness* should be the test of vitality, then enamel is only *vital* when it shows *sensitiveness* and when it does not show it, why it must be *dead*.

3. Sensitiveness is not one of the most certain signs of vitality wherever it (vitality) exists, nor wherever it (sensitiveness) exists, (sentence capable of each construction and wrong in each ;) a sporule of mildew, a grain of wheat or of corn, or any vegetable seed, may germinate, grow, &c., but is there any sensitiveness about this or about any vegetable growth ? and in the first few weeks of human embryonic life, when not an animal system has yet been evolved, but the organic life is active indeed, there cannot be sensitiveness for there is no nervous system, yet here is an abundance of vitality ; *so there may be vitality without sensitiveness.*

"*Extreme sensitiveness*," is nearly allied to *pain*, and Dr. Thos. K. Chambers of London, in his excellent lectures upon the "Renewal of Life" says. "Pain in short is the brother of death; a painful part is never performing its whole vital functions—it is partially defunct."

It is therefore the best evidence of some kind of lesion of the peripheral nerves or nerve centres and indicates disease; *pain*, *extreme sensitiveness*, and *sensitiveness* are attached to and depend upon the nervous system but have no relation save an incidental one to the functions of organic life.

Enamel has no nerves and usually no fibrillæ; dentine has fibrillæ which act as nerves; the sensitiveness of dentine depends upon its fibrillæ; if enamel should *ever* be "*extremely sensitive to the touch*," it is from a deranged condition of the *dentinal fibrillæ* accidentally prolonged into the enamel. Calcified Enamel and Dentine are dead.

It is with no spirit of hypercriticism that I have made the above defence—but to sustain and advance the opinions, which a most carefully study of the histology of the present day, justifies my holding.

---

